# Inoculation—Vaccination

**Published:** 1889-05

**Authors:** Frank S. Billings

**Affiliations:** Director of the Patho-Biological Laboratory of the State University of Nebraska; Lincoln, Neb.


### *INOCULATION—VACCINATION.*

By FRANK S. BILLINGS,

Director of the Patho-Biological Laboratory of the State University of Nebraska.

When one comes to discuss this question with the members of the profession as he casually meets them, he is often surprised to find how uncertain and clouded the prevailing ideas are upon the subject. In fact, the majority of physicians do not seem to have any clear conception of the act connected with the words "inoculation" and "vaccination."

### INOCULATION.

What is inoculation?

Inoculation is the transmission of a communicable disease from diseased to healthy individuals of the same species in which such a disease occurs naturally, or any other susceptible form of animal life, by means of the introduction of material from such diseased organisms in an artificial manner.

We may call that the intentional production of the disease in healthy individuals by diseased matter.

Inoculation may or may not have prevention as its definite purpose. When the latter is the ultimum of our endeavors, we have to consider several points before undertaking it.

1. Is such and such a disease extra- or intra-organismal in its primary origin? That is, is it exogenous or endogenous—to speak with Pettenkofer? (The words "contagious" and "infectious," as expressing class or differentiation, should be banned by all intelligent medical writers, as no one can tell the etiological nature of any infectious disease, unless personally acquainted with it from the writings of the average medical author. The bacteriological school is a blind leader of blinder followers in this regard.)

2. Given an infectious disease, is it recurrent or non-recurrent? Does one attack, be it severe or mild, give immunity through life to the individual diseased, as a rule?

3. This being the case, such a disease is absolutely preventible by inoculative treatment in some artificial form, whether we can at present command such a method or not. It will eventually be found true of that terror of childhood, scarlet, and those milder complications, measles, mumps, chicken-pox, etc., as well as typhus and the yellow fever, must finally yield to the results of scientific investigation. The opponents of actual vaccination should be confined in a lunatic asylum. They are asses of the most extremely long-eared type.

The first attempts at inoculation as a means of prevention were known as

### VARIOLATION.

It was first practised by the Chinese before the birth of Christ, but was not inaugurated in Europe until many centuries later. In variolation, the inoculator simply takes matter from the eruptions of a small-pox patient, and pricks it into the skin of a healthy person. Small-pox is the almost inevitable result. Why, then, was it done? There were two reasons. Anyone at all acquainted with the history of the world before this century, especially in the fifteenth, sixteenth, seventeenth, and eighteenth centuries, of which we have more full accounts than of those before that time, must know what terrible misery and desolation this plague caused. Many districts, and even cities and towns of considerable extent, were almost depopulated by it. Variolation was first undertaken to make the whole community sick at once, and thus put an end to the desolation as speedily as possible, for it was as well known then as now that those who recovered were seldom liable to a second attack.

Something more was known than this, however, or, perhaps, something better resulted from close observation or experience, for these ancient physicians were fully as close observers of symptoms and results as their more minutely instructed successors of to-day. These men soon learned two very essential facts:

*First*—That almost imperceptibly mild cases of the natural disease enjoyed the same immunity from a second attack of small-pox as those who recovered from it in its most malignant form.

*Second*—They found out that the well-known rule, "Like begets like," also held good in this case; or, in other words, that the inoculation (variolation) of healthy individuals from the most extremely mild cases generally resulted in the production of a similarly mild attack in those inoculated, and gave the same degree of protection to the individuals.

This was a tremendous discovery. All the mechanical money-making, money-saving inventions of the world bear no comparison to it in its value to the human race; but those terrible heathens, the Chinese, knew it centuries before the more enlightened Christians ever became aware of it. As a matter of history, it may be mentioned that its popularization in England was due to the nerve of a woman, an English duchess, who learned of the value of the procedure on the continent, and had her own children variolated, and successfully, thus leading the way to saving thousands of lives for her countrymen.

This variolation, however, had its dark side. Persons acquiring the disease in this way were as dangerous to healthy individuals as those who acquired the small-pox in the natural manner; hence, while extensively practised, and in many places made obligatory by law, as soon as small-pox broke out in a community, still the history of that time is full of edicts of how it should be done, and by whom, and requiring that every person that had not had the natural disease should be variolated; but the districts in which it was done were shut off from those where it was not done, by a strict quarantine, and the entire locality treated as if a natural outbreak were occurring, of which, indeed, this variolation was but an artificial extension or generalization. It is easily to be seen that such a procedure was not only more or less dangerous, but very disturbing to the comfort and well-being of a community, and, except with very courageous individuals, was only resorted to extensively at periods of great danger. Nevertheless, the accurate judgment of the doctors of the time was very great in the selection of those cases from which others could be variolated without danger to their lives. What great events often follow apparently insignificant observations! How many thousands pass heedlessly by a great natural phenomenon without ever giving a single thought to it! Few observe. Still fewer think.

### VACCINATION.

The word "vaccination" is from *vaccina*, which is again from the Latin word for cow, and means the transmission of what was called cow-pox, vaccina, to human beings. Cow-pox is nothing more nor less than small-pox, which, nobody knows when, had been conveyed to cows by milkers, either diseased themselves, or, what is more probable, busied in caring for diseased friends. Milk-cows have seldom perfectly whole skin on their teats, and so this unknown eruption, so far as its cause went, was continually being conveyed from cow to cow by the milkers, and finally acquired a regular constancy in weakness—from unknown causes also—in comparison to the human small-pox So long as there was an abundance of small-pox, there was also plenty of cow-pox, but as vaccination has gained the mastery over small-pox, so has the cow-pox disappeared, but to a much greater extent; for it is very doubtful if there has been a single case of real cow-pox in this country within the last twenty-five years. No better evidence of the value of vaccination can possibly be asked for than this indisputable fact. The cow-pox from which our present vaccine is obtained is the continued transmission of the original disease of years ago. The introduction of this great blessing into this country is due to one of the most learned and gifted physicians the world has ever known, the late Dr. Henry A. Martin, of Boston. People called him a "crank" when he lived. Now history blesses his memory. Were not the cow-pox the natural derivation of the small-pox, it would never have had any preventive value against the latter.

The latter fact became a matter of practical experience, which seems only to have been known to those connected with dairy cattle long before the medical world had any intimation of its value. Jenner learned it from the dairy-maids of Glostershire. Thus it is to be seen that woman again played the most important role in this second and even greater blessing to humanity. But Jenner had a fight. The word "crank" had not been coined then—thanks to the want of American vulgarity—but the "crank" of public opinion drew the rope of ignorant opposition most distressingly tight about the life of this world-wide benefactor, though his services were finally recognized by the British Government.

It took a long time for genuine vaccination to win its way. People were afraid of it, and everything which was possible was done to throw ridicule upon it. Even that great artistic genius, Hogarth, threw the influence of his characteristic abilities against it, and has left us a picture which well illustrates the popular prejudices of the time. It is a very singular fact that the very counterpart of the ideas pictured by Hogarth sprang into the minds of the boys I took over to Pasteur to be inoculated against rabies, of which they never were in any danger. The material used by Pasteur was made from the spinal cords of rabbits which, according to him, had had artificial rabies, but which I do not yet believe to be a fact. These cords were treated in a peculiar manner, and then rubbed up very finely in water, when a morphine syringeful of a portion of different cords was injected under the skin of the patients each day, leaving little bunches in each spot, which disappeared in the course of time. The boys noticed these bunches, and as the injections were made in the skin of the abdomen, they got to discussing the matter among themselves, and finally became anxious about it, and came to me one day in great distress desiring to know "if young rabbits would grow out of their bellies where them bunches were which old Pasteur had made?"

Vaccination, then, is still an artificial form of variolation. It is the direct introduction of the product of the disease into healthy individuals, but its great difference from pure variolation is that, in vaccination, the diseased material has been so mitigated in virulence that it produces a variola so mild as not to have much, if any, resemblance to the original. But what is of the greatest importance is, that the vaccinated person is not a source of danger to healthy or unvaccinated persons.

This might properly be called Jennerism.

We now come to a second and very important advance in preventive inoculation, which may very properly be termed

### PASTEURISM.

While I am a most decided opponent of the methods of M. Pasteur, and shall criticise them in what may appear a regardless manner, still I have a no less high appreciation of the value of his work to humanity. Pasteur may be said to be an explorer into an unknown sea. He is an indicator of directions, not a completer of methods. Leaving his anti-rabies inoculation entirely out of the question, as I do not believe in it at all at present, though evidence may yet be furnished which will cause me to change my opinions, I will confine myself to those methods of preventive inoculation which have far more interest at the present time. I mean the preventive inoculation with regard to the infectious blood-poison diseases, which includes hen cholera anthrax, black leg, and the rouget or erysipelas of the hog, a disease at present not known to exist in this country, but which causes serious losses to the farmers of Europe.

What is Pasteurism? Pasteurism differs essentially from Jennerism in that, instead of introducing the products of disease in any form into healthy individuals in order to give them immunity to a natural attack of a given disease by producing an almost imperceptibly mild affection, we endeavor to produce the same result by the introduction of the specific cause of the disease, but so mitigated in an artificial manner in its virulence, that there is no danger to the life of the individual thus inoculated.

It has become a generally accepted fact that all infectious diseases are due to a specific cause, which belongs to some specific species of microscopic vegetable life. The scientific class to which these little objects belong is the fungi, their special name being bacteria, or, in common parlance, germs. It is not essential to go into any description of the various varieties of these germs in an article of this kind, still it is necessary that we know a little something of the manner of action of that class in which we are momentarily especially interested. In the diseases of animals Pasteur has been, beyond question, successful in preventing natural infection by his method of inoculation, the names of which have already been given, and to which may be added swine plague and Texas fever in cattle, as well as the yellow fever and typhoid fever in man, all of which are forms of septicemia, or blood poisons; the specific poison in each case being the direct result of certain unknown physiological actions on the part of the specific germ, which causes each of these diseases. All these diseases have also another attribute in common, viz., they are extra-organismal in origin; that is, the place of primary development of the specific germ, which causes each of these diseases, is outside the animal organism, or in the earth or earthy material.

PASTEUR'S METHODS.

The fact that the specific cause of these diseases (the prevention of which Pasteur has shown to be possible by his methods,) finds its place of primary development and continuous existence outside the organisms of those species of animal life in which such diseases occur as a natural infection, seems never to have impressed itself upon the mind of Pasteur. It should be stated that Pasteur has little or no knowledge of disease, and hence is absolutely incapable of coming to any clear comprehension of the true nature of any disease. In this fact is to be sought the reason that he has permitted his methods to be generally used in France, with utter disregard of the necessary precautions, and his absurd attempt to clean out the rabbits in Australia by sewing hen cholera germs in the grass, a disease which does not infect rabbits under natural conditions. Had Pasteur appreciated the true place where the germs of these diseases originally live, and from whence they gain access to a susceptible animal organism; had he, in fact, been a pathologist, no condemnation of his methods or principles could be too severe. But as he does not know these things, we will pardon what, in others, would be gross criminality, and give him credit for the great work he has inaugurated—a work which, eventually, will far exceed that of Jenner in its value to humanity.

Pasteur recognized the fact, which no one of any experience will dispute, that there are many diseases in which even a mild attack renders the individual insusceptible to a second as a general rule.

Pasteur also knew another fact, that the diseases we have previously spoken of, as well as all others of a similar nature, were, or are, due to some specific form or germ life, and though he has never, to my knowledge, stated it, still I think he should be credited with recognizing the fact that cow-pox was but mitigated small-pox, rendered, as has been said, non-malignant in character by successive generations of transmission from cow to cow in an accidental and unintelligent manner. Pasteur endeavored to do this same thing intelligently. He but repeated the lesson thus learned in his first attempts at mitigating the action of the germs of hen cholera, anthrax and Rothlauf, by experimental inoculation upon various species of animals; and he found that while in some species a given germ acquired even more virulence than it possessed when taken from an animal in which it had caused the natural disease, and kept on increasing in virulence for a time as he passed it from animal to animal of a certain species, still, on the other hand, the same procedure in another species of animal, carried on through a long series, not only caused the germs to lose in virulence, but after a time they acquired a certain standard of mildness, and could be safely used for inoculation.

This was imitating the results in small-pox. Pasteur went further. Such a method as the above is open to the very serious objection that not only is an almost endless number of experimental animals necessary, but the expense would finally be such as to decidedly interfere with any practical benefits resulting to humanity. This fact led Pasteur to seek similar results in an entirely different direction. He and others have experimented in many ways to obtain this result, but the continued exposure of artificial cultivations of given germs to certain constant degrees of temperature, has given the best and most trustworthy results; and what is most singular of all, when once a certain desired constancy of mitigated virulence in some germs has been obtained, it has been found that successive cultures can be then carried on for a very long period at the ordinary room temperature, and that each culture will have the same degree of mitigated virulence as the previous one. This enabled Pasteur to prepare what he calls a primary or weak vaccine, and a secondary or strong. The results following upon the practical application of Pasteur's method have been quite flattering, but, notwithstanding the fact, they must be absolutely condemned, and should be forbidden by law, unless governments prepare inoculation stations, where all danger of local infection can be absolutely nullified, or those doing such work take similar precautions.

### OBJECTIONS TO PASTEUR'S METHOD.

It has been stated that the diseases mentioned are all of extra-organismal origin; that is, their specific germ finds its place of local development in the earth or earthy surroundings of animal life.

No matter in which way we may follow Pasteur in the preparation of mitigated material for preventive inoculation, we have still to do with the fact that that material always contains the specific germs of the disease, originally capable of producing it in a malignant type when it first entered the animal from which the primary culture was taken.

This is another fact never properly appreciated by Pasteur.

We must now return to the first statement, viz.: That the primarily infected organism derived the germs causing the disease in question, let it be anthrax, chicken cholera, swine plague, or yellow fever, from its surroundings, or in food, or from material grown or derived from such localities. By the use of Pasteur's method, we invariably introduce the germ into the inoculated organism, and this germ finally finds its way into the intestines, and from thence again to the earth, which is its natural abiding place, and here, if the locality offers the desirable nutrient conditions, it again acquires its original virulence and again becomes dangerous to susceptible species of animal life. This statement cannot be disputed. The germs of anthrax, yellow fever, Texas fever, Rothlauf, the German Wild-seuche, typhoid fever, swine plague, and other such diseases, all live in the soil; and it is so much my opinion that in certain soils these germs live in "innocuous desuetude"—that is, that originally they are absolutely non-malignant, and that they owe their virulence to the addition of the chemical refuse of animal life, such as manure and urine, to those soils in which they are then forced to live, and from which they acquire a new, as we call it, virulent activity, and become thus malignant and disease-producing to certain species of animal life—that I am now preparing the means to endeavor to demonstrate this fact by actual experiment. In fact, I have already partially demonstrated this point by experiment.

In October last, I inoculated thirty healthy hogs with a virus so weak that even the closest observation could scarcely perceive any disturbing effects in any of these animals, but a healthy (uninoculated) pig which was placed among the lot succumbed to swine plague in course of time. It might be objected that this hog became infected from the pen, but that is impossible, as the hogs were placed in a new building with brick walls and an asphalt floor, where no hog had ever been; and, again, had the germs been there previously, some of the inoculated animals would have succumbed, but this animal did not die until the danger to the inoculated pigs was fully over, showing that the inoculation had had time to protect them, while the germ was so increasing in virulence in the manure on the floor that it eventually caused the infection of the uninoculated animal. This is but one experiment, but sufficiently conclusive to have a warning value, especially as it is most emphatically supported by more extensive results of the same nature recorded by Schottelius and Lydtin, in Germany, with regard to Pasteur's vaccine against rouget, where some healthy pigs got to the manure pile taken from the pens of the inoculated ones, and acquired the disease. I have repeatedly found the germs of anthrax in the manure passed by cattle having that disease; in fact, in one quite alarming outbreak to which I was called at Sioux City, Ia., I made my microscopic diagnosis in the first case in this way, and afterwards confirmed it by the examination of the blood of the animal after the autopsy had been made.

This, then, is a sufficient reason why we cannot apply the present method of preventive inoculation to swine plague, except when done by very trustworthy persons and under most exact isolation precautions. That it will prevent, has been so placed beyond all question by the testimony of some of the best-known and trustworthy farmers and swine-breeders of Nebraska, and my own exact experimentation at the station, that no reasonable and unprejudiced person can for a moment doubt the establishment of the fact.

Artificial inoculation is but the endeavor to imitate the results occurring in natural infection. If, in any given disease, a mild attack produces a condition of immunity toward a second, upon sufficient exposure to infection, anyone of common sense should know that, if we can artificially follow nature in this regard, we can produce a similar immunity by inoculation. It is the universal testimony that such immunity is the rule in swine plague under natural conditions. It is well known that I have the germ of swine plague in my possession, and equally well known that I have produced not only the malignant type, but have successfully imitated nature, by producing a mild and non-fatal type of the disease by inoculations with pure cultures of the germ. This thing has been demonstrated time and again, and, as said, testified to by practical farmers of undoubted repute, so that it must be looked upon as an established fact that swine plague can be prevented by inoculation.

### THE METHOD.

Had I followed Pasteur's methods, I certainly should not publish it here, for I consider their further extension, or any knowledge of them, except to the reliable experimentalist, can only result in bringing a possible blessing to humanity into disrepute, and end in bringing a still greater amount of evil upon an already sufficiently afflicted portion of the community.

The method employed by me, in establishing the fact of preventive inoculation, though the one at present pursued is far more complicated, is simply the result of long-continued practical observation and protracted and exact reflection upon all the varied phases and phenomena of swine plague. It is an exact imitation of nature. It might be styled porcination. I had seen a very large number of outbreaks of the disease in which but a very few animals died, and in which the course of the disease was unusually protracted, and especially in which there was an absence of any hemorrhagic lesions in the lymph glands or other organs of the body. The more pneumonia, and the more chronic its type, the more button-like neoplasms, or growths in the intestines, the better. I had also seen that the balance of such a herd would remain entirely well, even when newly-purchased pigs from uninfected localities happened to be put where these pigs were and soon died in large numbers from the disease in its malignant form. Numerous cases of this kind coming to my notice, I began to inquire diligently, of the most intelligent farmers, as to their experience, and found that they had made the same practical observations, but were totally unable to explain them satisfactorily to themselves. I then called their attention to my ideas upon the matter, and excited their interest, and have repeatedly received unquestionable confirmation of their correctness.

The next step was to repeat nature's work. Selecting two sick hogs, one from a very malignant, acute, and excessively hemorrhagic outbreak, and the other from one of the kind previously mentioned, exactly contrary, I made fluid cultures from the spleens of each, and, after satisfying myself of their purity, I inoculated healthy pigs with exactly the same amount of material, in the thin skin of the inside of the thigh. In the first case, all were sick, and the majority died. In the second, none died, the only disturbance being that the animals were "off their feed" for a few days, and were somewhat disturbed in deportment, their bristles being a little roughened, and they were less lively than usual. Following the latter method, I have never lost a hog from preventive inoculation, and have acquired sufficient skill in the matter to be able to select the outbreak and animal from which one inoculation of one c. cm. of a fluid culture can be depended upon to produce as reliable a degree of immunity as that which occurs under conditions of natural infection.

Although I have perfected a method of cultivation by which it is impossible to keep cultures for inoculation at any desired degree of virulence, and, apparently, for an indefinite period, still it is my firm conviction that, in order to arrive at a practical solution of this problem with regard to the entire class of non-recurrent exogenous diseases, that we must eventually have recourse to the chemical method. As to the endogenous diseases,

I do not think any results will ever be attained in this way, but that in all cases preventive inoculation will have to be made with material in which the germ is suspended, or upon which it is bound, though mitigated in virulence, even as we now see it in vaccina from the cow. The only question being, Can it be done artificially and with pure cultures of the etiological organisms without recourse to animal life, in order to retain or refresh the constancy of virulence ?

### THE CHEMICAL OR PTOMAINE METHOD FOR THE PREVENTION OF NON-RECURRENT EXOGENOUS DISEASES BY INOCULATION.

It has been said that this group of diseases are all blood poisons, or, in other words, the disease itself, while caused by its specific germ, it is still through a product of that germ, while within the organism, which is constantly added to the blood, that the disease is generated. In this regard, the group of endogenous diseases is more or less sharply distinguished from those of an exogenous nature. In the former we either find a characteristic eruption, or a specific organic lesion, which is that peculiarity by which we recognize their existence, while in exogenous diseases it is more the general picture which is specific, and the lesions called characteristic are dependent upon the physiological and anatomical peculiarities of the organism diseased rather than upon the action of the germs ; yet many of these organisms also develop some striking symptom of action by which we recognized the disease induced, when taken in connection with the whole picture, but not pathognomonic of itself, as icterus in yellow fever, which gives it its name, but that is a general symptom rather than a local eruption or organic complication. It is at present a matter of doubt, in my mind, as previously noted, whether the endogenous diseases can ever be prevented by any product of their specific germs, or, in other words, though we may yet be able to treat them in this sense by pure and mitigated cultures, still I think the germ itself will always have to be used where such diseases are open to preventive inoculation. But, as in vaccination, we must be able to so change the virulence of the germ that, while we induce a mild type of the disease capable of producing immunity, still the inoculated individuals will not be dangerous to healthy ones. It must always be remembered that in endogenous diseases it is the diseased individual that is directly dangerous to healthy ones, through the breath, or the direct product of the disease; that is, something from the diseased body. In exogenous diseases this is not the case; it is the excreta, the natural effete material, which is dangerous, but not directly; it must first reinfect, or infect earthy surroundings, or as such, gain direct entrance in some accidental manner to healthy individuals. Contact, as we call it, with diseased individuals is never sufficient of itself to endanger healthy ones.

This latter class are all due to a poisonous principle generated by their germs, and added to the blood. It should be self-evident, from all which has been said, that we must not use any material containing the germs of such diseases for preventive inoculation, except with great care, because those germs will be passed off in the excreta of the inoculated individual; and outside materials being their natural home, we are thus continually sowing malignant seeds which may, at any time, become a source of danger to healthy individuals. On the other hand, we know that the germs produce a poison, and that this poison produces the real disease. I have seen this in swine plague. I have even killed a healthy pig with all the symptoms of swine plague in life, and all the genuine lesions of the disease in death, by the continued daily introduction of a given amount of a fluid culture of the germs of a very malignant type, but in which the germs had all been securely killed by heat, their sure death being proven by negative attempts at new cultures from that material. In the organs of the dead hog not a germ could be found. I have also produced complete immunity in the same way, and have quite a number of pigs at the station, which are swine-plague proof, that in their preparation never received a germ of swine plague. This method is not practical, however. It is only the necessary step toward the chemical method. This is a work of slowly developed stages. First, we have to demonstrate that immunity can be produced by the germs themselves when mitigated in virulence. Second, by the material in solution without the germs. This, however, takes too great a quantity of material ever to have even the apparent practical value of the first method An illustration will at once show these conditions very forcibly.

Quinine, as a remedy in chills and fever, comes as near being a specific as anything known in medical practice. Quinine is the alkaloid, the active principle, in Peruvian bark. Were any person to endeavor to attain the same effects in this disease by taking the decoction made by the prolonged soaking of the bark in water, which he can obtain from two or three grains of quinine, in order to introduce these two or three grains of quinine into his system, he would have to drown himself, inwardly, with the decoction, and probably become almost sick, in another way nauseated, before he could do it.

Hence, in order to get at a practical method of preventing such diseases as the yellow fever, typhus, or the swine plague, we must turn the business over to the chemist, if we ever hope to obtain a material which can always be reliable, always have the same composition, which will keep well, and, above all, of which we can fix the dose and have it in the smallest, most compact, and, at the same time, concentrated form. In other words, we must extract the active principle (the quinine) from the great volume of fluid in which it has been developed, and in which it is in solution. This active principle of germ life has already been chemically reduced in connection with a few germs. This is a new method of research. It is entering almost blindly into a dark wilderness of unknown facts. Dark as it is, still it promises more for humanity than anything which is open to the energies of scientific investigation. Public health is public wealth, and animal health is a tremendous factor in our public health as well as national prosperity. The pathologist cannot do this work. He can only go so far as to show that it can be done, and aid and control the work of the chemist in perfecting it, for even then the work of animal experimentation of the practical demonstration of the fact of ptomaine prevention belongs to the pathological investigator.

LINCOLN, Neb., April 1, 1889.

